# Acaricide resistance among single-host and multi-host ticks in sub-Saharan Africa: Current status, drivers, and control strategies

**DOI:** 10.14202/vetworld.2026.1681-1690

**Published:** 2026-04-28

**Authors:** Mandla Yawa, Nkululeko Nyangiwe, Luxolo Qokweni, Zamantungwa T. H. Mnisi, Zuko Mpisana

**Affiliations:** 1 Döhne Agricultural Development Institute, Stutterheim, South Africa; 2 Department of Agriculture and Animal Health, University of South Africa, Florida, South Africa; 3 Department of Agricultural Sciences, Nelson Mandela University, South Campus, Gqeberha, South Africa.; 4 Department of Agriculture, Gqeberha, South Africa; 5 Department of Biodiversity, DSTI-NRF SARChI Chair (Ecosystem Health), University of Limpopo, Private Bag X1106, Sovenga, 0727, South Africa; 6 Department of Livestock and Pasture Science, University of Fort Hare, Private Bag X 1314, Alice 5700, South Africa.

**Keywords:** acaricide resistance, biological control, climate change, integrated tick management, multi-host ticks, single-host ticks, sub-Saharan Africa, tick-borne diseases

## Abstract

Ticks and tick-borne diseases (TBD) continue to pose a significant threat to livestock production and rural livelihoods in sub-Saharan Africa (SSA). The control of ticks has relied predominantly on the use of chemical acaricides; however, the widespread and often indiscriminate application of these compounds has led to the rapid emergence and spread of acaricide resistance. This review provides a comprehensive synthesis of current knowledge on acaricide resistance in both single-host ticks, such as *Rhipicephalus microplus* and *Rhipicephalus decoloratus*, and multi-host ticks, including *Amblyomma variegatum* and *Rhipicephalus appendiculatus*, across tropical and subtropical regions of SSA. The evidence indicates that resistance is more pronounced in single-host ticks due to their continuous exposure to acaricides throughout their life cycle, whereas multi-host ticks exhibit emerging resistance patterns influenced by intermittent exposure and ecological adaptability. Multiple resistance mechanisms, including target-site mutations, metabolic detoxification, and behavioral changes, contribute to reduced acaricide efficacy. In addition, environmental factors such as temperature, humidity, vegetation, and climate change play a crucial role in shaping tick distribution, population dynamics, and resistance development. Misuse of acaricides, lack of proper rotation strategies, and limited farmer awareness further accelerate resistance emergence, particularly in smallholder farming systems. The review highlights the urgent need for sustainable and region-specific control strategies. Integrated Tick Management (ITM), combining chemical rotation, biological control agents, pasture and habitat management, and farmer education, offers a promising approach to mitigate resistance and improve tick control outcomes. However, gaps remain in understanding resistance patterns in multi-host ticks and wildlife reservoirs, as well as in the implementation of coordinated surveillance programs. Strengthening research, improving stakeholder collaboration, and enhancing farmer training are essential to ensure the long term effectiveness of tick control strategies and to safeguard livestock productivity in SSA.

## INTRODUCTION

Ticks and tick-borne diseases (TBD) remain a major constraint to livestock production in sub-Saharan Africa (SSA), threatening both animal health and rural livelihoods. As obligate hematophagous ectoparasites, ticks transmit a range of protozoal, bacterial, and viral pathogens responsible for economically devastating diseases, including East Coast fever (*Theileria parva*), babesiosis (*Babesia bovis* and *Babesia bigemina*), and anaplasmosis (*Anaplasma marginale*) [1, 2]. These infections collectively impose annual economic losses estimated at USD 22–30 billion on the livestock sector, with smallholder farmers disproportionately affected due to their reliance on cattle for food security and household income [3]. Among these diseases, East Coast fever stands out as the most lethal and economically significant, causing mortality rates approaching 100% in naïve herds and chronic productivity losses in survivors, including reduced milk yield, weight gain, and fertility [2]. Similar burdens are reported for bovine babesiosis and anaplasmosis, which remain endemic across much of Eastern and Southern Africa and further compound management costs [4].

The epidemiology of TBD in SSA is strongly shaped by ecological and climatic factors. Warm, humid environments such as the Great Rift Valley and coastal zones support year-round tick activity, whereas climate change is expanding tick habitats into previously marginal areas and higher altitudes, intensifying disease risk [5]. Tick population dynamics are further influenced by livestock density, vegetation type, and seasonal rainfall, which together determine the frequency of acaricide use and the likelihood of acaricide resistance development [6]. Over the past decades, chemical acaricides, including synthetic pyrethroids, organophosphates, and amidines, have remained the cornerstone of tick control. However, the heavy and often indiscriminate application of these compounds has driven widespread resistance in key vectors such as *Rhipicephalus microplus*, *Rhipicephalus decoloratus*, and *Rhipicephalus appendiculatus* [7, 8].

Resistance evolution is particularly pronounced in single-host ticks, whose life cycles on a single animal expose them continuously to chemical treatments, thereby accelerating selection pressure. In contrast, multi-host ticks such as *Amblyomma variegatum*, *R. appendiculatus*, and *Hyalomma* spp. experience intermittent exposure, yet their ecological adaptability and capacity for cross-resistance pose emerging challenges for control [9]. Geographic “hotspots” of resistance are now documented across southern, eastern, and western Africa, often coinciding with communal farming systems where acaricide misuse, limited product rotation, and high livestock densities create ideal conditions for the selection of resistance [9, 10].

The accelerating spread of acaricide resistance threatens not only livestock productivity but also the efficacy of current tick-borne disease control programs. The growing evidence of climate-driven tick range expansion, coupled with intensifying selection pressure from chemical control, underscores the urgent need for sustainable, region-specific management strategies. Integrated tick management (ITM), combining acaricide rotation, biological control, resistant livestock breeds, and environmental interventions, offers a promising alternative but remains underutilized across SSA [4, 11].

This review provides an updated synthesis of acaricide resistance in both single- and multi-host ticks in tropical and subtropical regions of SSA. We explore the mechanisms of resistance, regional patterns, and environmental drivers, identify critical knowledge gaps, and highlight opportunities for implementing ITM strategies to safeguard livestock production and rural livelihoods in a changing climate.

## REVIEW METHODOLOGY

A comprehensive literature search was conducted using electronic databases including PubMed, Scopus, Web of Science, Google Scholar, and African Journals Online. The search covered peer-reviewed articles, reports, and dissertations published between 2010 and 2025 using combinations of the following keywords: “acaricide resistance”, “Rhipicephalus microplus”, “Rhipicephalus decoloratus”, “Amblyomma variegatum”, “Rhipicephalus appendiculatus”, “single-host ticks”, “multi-host ticks”, “sub-Saharan Africa”, “tick-borne diseases”, and “integrated tick management”. Additional relevant publications were identified through manual screening of reference lists from retrieved articles. Only studies reporting acaricide resistance data in cattle ticks from tropical and subtropical regions of SSA were included. Non-English language articles, conference abstracts without full text, and studies focused exclusively on non-cattle hosts were excluded. Data extraction focused on resistance status, mechanisms, geographic distribution, environmental drivers, and control strategies. A total of 45 relevant sources were synthesized narratively to provide an updated overview of the current status, drivers, and sustainable control options.

## GEOGRAPHICAL PATTERNS OF ACARICIDE RESISTANCE IN SINGLE-HOST AND MULTI-HOST TICKS

In SSA, the geographical distribution of acaricide resistance among single-host ticks (*R. microplus*) and multi-host species (*R. appendiculatus* and *A. variegatum*) reflects complex interactions among environmental conditions, tick ecology, and acaricide application practices. Widespread acaricide resistance has been documented across Southern Africa, with strong evidence from communal farming systems in South Africa indicating that *R. microplus* populations exhibit high and increasing resistance to deltamethrin (C_2__2_H_19_Br_2_NO_3_), a synthetic pyrethroid acting on voltage-gated sodium channels, and to amitraz (C_19_H_2__3_N_3_), a formamidine that targets octopamine and tyramine receptors in ticks. Resistance has also been reported to other synthetic pyrethroids, including cypermethrin, cyfluthrin, and alphacypermethrin, as well as to additional acaricide classes such as organophosphates (e.g., chlorpyrifos, coumaphos) and macrocyclic lactones (e.g., ivermectin). These patterns reflect the involvement of multiple resistance mechanisms, including target-site mutations and enhanced metabolic detoxification [12]. In West Africa, for instance in Côte d’Ivoire, resistance against deltamethrin and amitraz has also been reported in *R. microplus*, indicating that the problem is not confined to southern regions [13].

Multi-host ticks show somewhat different patterns: in East and West Africa, *A. variegatum* and *R. appendiculatus* stocks have shown medium to high levels of resistance to certain acaricides, though often less intense than *R. microplus* [6, 12]. Geographic hotspots of resistance often coincide with high livestock density, frequent acaricide application, and environmental conditions like warmer and more humid climates that favor tick survival and reproduction. Therefore, understanding these patterns regionally is essential for designing management strategies that are tailored to ecology, climate, and local acaricide use practices.

## DISTRIBUTION AND PATTERNS OF ACARICIDE RESISTANCE

**Figure 1:**
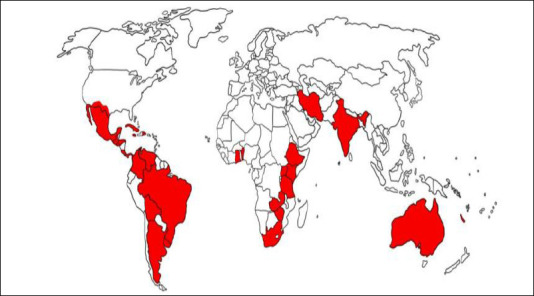
Global geographic distribution of acaricide resistance of single-host ticks [6].

*R. microplus* exhibits high levels of resistance to pyrethroids in South Africa and Côte d’Ivoire, while multi-host ticks such as *A. variegatum* and *R. appendiculatus* show lower but emerging resistance in parts of East and West Africa. Single-host ticks, including *R. microplus* and *R. decoloratus*, are widely distributed across SSA, particularly in East and Southern Africa, where cattle production is intensive (Figure 1). Evidence from several studies indicates substantial acaricide resistance in *R. microplus* populations in countries such as South Africa, Zimbabwe, and Kenya, largely driven by frequent and often unregulated acaricide use, which increases selective pressure [14]. The distribution and intensity of resistance in single-host tick species are further shaped by management practices, host availability, and climatic conditions that support persistent tick populations. Warm temperatures and seasonal rainfall in East and Southern Africa enable year-round tick survival, often necessitating frequent acaricide applications. This repeated exposure has contributed to widespread resistance, particularly to synthetic pyrethroids and organophosphates [15]. Moreover, in arid and semi-arid regions where livestock congregate at limited water sources, high animal densities create localized “hotspots” of acaricide exposure, accelerating the emergence and spread of resistance [3].

Multiple-host ticks, including *A. variegatum*, *R. appendiculatus*, and *Hyalomma* spp., exhibit distinct resistance patterns influenced by their life stages, which involve multiple hosts and habitats. Several studies indicated that Ixodid ticks, such as *Amblyomma*, *Hyalomma*, *Rhipicephalus*, and *Boophilus* subgenus, prefer cattle as the host. These ticks are widely distributed across SSA, ranging from humid coastal zones to semi-arid savannas. Unlike single-host ticks, multi-host ticks encounter acaricides intermittently, depending on host interactions and environmental factors, which reduces direct selection pressure. However, their adaptability and cross-resistance to different acaricides pose a challenge to effective control [1, 2].

Regional variability in resistance among multi-host ticks appears related to climatic and ecological conditions. For instance, *R. appendiculatus*, the principal vector of East Coast fever, is predominantly distributed in humid and moderately temperate high-rainfall regions of Kenya, Uganda, and Tanzania, where environmental conditions favor tick persistence and pathogen transmission; acaricide resistance has been documented in populations from Uganda and neighboring areas [12]. These conditions allow ticks to thrive across all life stages, creating opportunities for resistance development in areas with consistent acaricide exposure [15]. In contrast, in the arid regions of the Sahel, multi-host ticks such as *Hyalomma* spp. and, less frequently, *R. appendiculatus* are relatively more resilient to extreme heat (temperatures approaching 40°C), dry conditions, and sparse vegetation. Under these conditions, tick burdens tend to be lower, which tends to reduce the frequency of acaricide applications. For effective implementation of ITM strategies, it is therefore essential to understand tick species’ geographical distribution, how changing climate and environmental variables impact tick survival and behavior, and how these factors influence the development of acaricide resistance [14].

## INFLUENCE OF ENVIRONMENTAL CONDITIONS ON ACARICIDE RESISTANCE

Environmental conditions, including climate, vegetation, and altitude, play a crucial role in the geographical distribution of acaricide resistance among tick populations. Temperature, humidity, vegetation, and altitude collectively shape tick survival, acaricide use frequency, and resistance evolution, with climate change expanding habitats into higher altitudes. Temperature and humidity have a direct influence on tick survival, reproduction, and activity, which, in turn, impact the frequency of acaricide application. For instance, high temperatures, moisture, and seasonal rainfall in East Africa and parts of Southern Africa promote tick activity and necessitate frequent acaricide use, contributing to resistance buildup in both single- and multi-host tick species [15].

Vegetation types also affect tick habitat suitability, influencing species distribution and resistance patterns. Adult *A. variegatum* ticks can be found in a wide variety of habitat types ranging from dense vegetation cover, moist savanna region and open grassland. However, *A. variegatum* nymphs are frequently encountered in shade than open vegetation habitats. In regions with such vegetation habitats, such as the forests and savannas of West and Central Africa, multiple-host ticks like *A. variegatum* thrive well. The dense cover provides ideal conditions for tick survival, particularly during nymphal stages, and the varied host availability in these regions promotes multi-host tick persistence [4]. Conversely, in humid and warm conditions of Southern Africa, single-host ticks such as *R. microplus* dominate, where frequent acaricide applications to livestock have driven strong resistance patterns.

Altitude also influences tick species distribution and acaricide resistance. Higher-altitude regions, like those found in parts of Kenya and Ethiopia, are generally less favorable for ticks due to lower temperatures, lower humidity, host availability and vegetation types; however, climate change has been linked to an altitudinal range shift for ticks, increasing the likelihood of resistance spreading into previously unaffected areas [10]. These shifting geographical boundaries indicate that environmental conditions and changing climate are dynamic drivers of acaricide resistance.

## IMPLICATIONS FOR REGION-SPECIFIC TICK MANAGEMENT

The regional variability in acaricide resistance necessitates tailored tick control approaches to account for local environmental conditions and tick species compositions. Climate factors, seasonality and land use are important factors for region-specific management as they impact patterns of tick distribution and resistance. In high-density cattle regions of East and Southern Africa, rotating acaricide classes and incorporating non-chemical control methods can help mitigate resistance buildup for single-host ticks [16].

Pasture management and biological control measures can contribute to reducing tick populations across both single-host and multi-host tick species by disrupting off-host survival stages and lowering overall infestation pressure. Practices such as pasture rotation and strategic resting reduce host availability for questing ticks and can limit tick survival irrespective of host-use strategy, thereby decreasing reliance on chemical control [14]. Rajakaruna and Eremeeva [17] indicated that roughly 85% livestock farmers do not practice acaricide rotation which worsens challenge of tick resistance. In areas where multi-host ticks are prevalent, integrating pasture rotation and biological control measures, such as natural predators, can reduce tick survival across diverse hosts and limit the need for chemical interventions [4]. Study conducted by Singh *et al*. [18] emphasized the importance of birds like oxpeckers (*Buphagus* spp.) to control tick infestation from large animals and that could lead to reduce frequency of acaricide application. However, birds’ effectiveness to control tick abundance highly relies on host availability and environmental condition of the region.

To effectively address acaricide resistance, surveillance and monitoring efforts are essential to identify resistance hotspots and tailor interventions. Mapping resistance patterns at regional level provides critical information to guide acaricide use and manage resistance. Incorporating climate modeling can further aid in predicting shifts in tick distribution, allowing for proactive management as climate shifts alter tick habitat suitability [12]. Involvement of agricultural institutions, veterinary pharmaceutical companies, non-governmental organizations and research institutions to provide technical support, awareness, training, infrastructure and implementation of ITM approaches for specific region is essential for sustainable tick control.

## CHALLENGES OF ACARICIDE RESISTANCE FOR SINGLE-HOST AND MULTI-HOST TICKS

Acaricide resistance is a critical challenge in the control of tick populations, particularly in SSA, where single- and multi-host ticks affect both livestock and human health. The misuse and over-reliance on acaricides, combined with limited alternative control options, have led to widespread resistance, undermining efforts to manage TBD effectively [2, 12]. This resistance complicates management strategies for both single-host ticks, such as R. microplus, and multi-host ticks, such as *A. variegatum* and *R. appendiculatus*, due to their distinct life cycles and ecological adaptations. Single-host ticks are prone to developing resistance due to their life cycle being spent on one host, leading to frequent and consistent exposure to the same acaricide, while multi-host ticks present challenges related to their complex life cycle and environmental survival which allow them to evade consistent chemical exposure.

## MECHANISMS AND DRIVERS OF ACARICIDE RESISTANCE

Acaricide resistance in ticks is driven by various mechanisms, including target-site mutations, metabolic detoxification, and behavioral adaptations. Single-host ticks, such as *R. microplus*, which complete their lifecycle on a single-host, are often heavily exposed to chemical treatments applied directly on livestock. This continuous exposure increases selection pressure, accelerating resistance development [16]. Research highlights genetic mutations in sodium channels associated with resistance to pyrethroids, as well as mutations in the acetylcholinesterase gene linked to organophosphate resistance [13]. Metabolic resistance, involving enhanced activity of detoxification enzymes such as esterases, also contributes to resistance in both single- and multi-host tick species [11].

In contrast, multi-host ticks such as *A. variegatum* infest different hosts at successive life stages, resulting in variable and often intermittent exposure to acaricides. Although this pattern may slow resistance development relative to single-host ticks, it poses substantial challenges for control, as each life stage is exposed to distinct environments and management practices [6]. Moreover, the high genetic plasticity of multi-host ticks facilitates the development of cross-resistance to multiple acaricide classes, particularly in regions where environmental acaricide contamination is widespread [19].

## ECONOMIC AND HEALTH IMPLICATIONS OF ACARICIDE RESISTANCE

The economic impact of acaricide resistance is profound, especially for smallholder farmers in SSA, who rely heavily on livestock for income and subsistence. Acaricide resistance in single-host ticks such as *R. microplus* often results in increased treatment costs and loss of livestock productivity due to recurring infestations [12]. Additionally, multi-host ticks contribute to the persistence of diseases such as East Coast fever and babesiosis, heightening livestock mortality and productivity losses. For example, resistant populations of *R. appendiculatus* have been linked to the spread of East Coast fever, with an annual economic burden estimated at $168 million in East Africa alone [3].

Ticks and diseases they transmit pose a major challenge to animal and human health. Crimean-Congo haemorrhagic fever is a zoonotic disease transmitted by Hyalomma spp. Resistance development remains a critical challenge in acaricide use on both single-host and multi-host ticks. Mtshali *et al*. [20] reported the resistance of multi-host tick *Hyalomma* spp. to deltamethrin and cypermethrin. A rise in resistant tick populations increases the risk of disease transmission to humans, particularly in areas with close human-animal interactions [21]. The increase in human cases of TBD such as Crimean-Congo haemorrhagic fever in Africa could be attributed to ineffective tick control, exacerbated by resistance [3].

## LIMITATIONS OF CURRENT ACARICIDE-BASED CONTROL STRATEGIES

Despite their widespread use, acaricides remain increasingly constrained by issues of accessibility, affordability, and declining effectiveness, particularly among smallholder farming systems in SSA. Many small-scale farmers have limited access to a broad spectrum of acaricide classes and therefore depend heavily on a small number of readily available products. Repeated and often unregulated use of the same acaricides exerts strong selection pressure on tick populations, accelerating the development and spread of resistance. This challenge is further compounded by limited farmer awareness of appropriate dosing, application intervals, and acaricide rotation principles, which undermines treatment efficacy and promotes resistance development [22]. In the case of single-host ticks, such as *R. microplus*, the continuous exposure of all life stages to acaricides on a single-host amplifies selective pressure, leading to rapid resistance buildup. Effective management of these species often requires frequent rotation among acaricide classes; however, this strategy is frequently impractical for smallholder farmers due to the high cost of products and limited market availability [23]. As resistance increases, farmers may respond by applying higher doses or shortening treatment intervals, practices that further exacerbate resistance and increase production costs.

Resistance dynamics in multi-host tick species are influenced by their ability to utilize multiple hosts and diverse environments, which can reduce uniform acaricide exposure and allow resistant individuals to survive and reproduce [22]. These ticks can persist in wildlife reservoirs or off-host habitats where acaricide exposure is minimal, complicating control efforts and facilitating the spread of resistant populations across landscapes. Environmental conditions, particularly high temperatures and humidity that support year-round tick activity, further intensify the need for frequent acaricide application, increasing selective pressure and accelerating resistance development [24]. Collectively, these limitations highlight the unsustainability of reliance on chemical control alone and underscore the urgent need for ITM approaches. Such strategies should combine judicious acaricide use with farmer education, resistance monitoring, biological control options, and improved husbandry practices to ensure long term effectiveness and sustainability of tick control programs [23].

## THE NEED FOR ITM APPROACHES

The rise of acaricide resistance in tick populations has led to the development of ITM strategies. These strategies combine chemical, biological, resistant cattle breeds such as Nguni, and environmental control methods to effectively manage tick populations while minimizing resistance. In SSA, where TBD significantly affect livestock health and productivity, effective implementation of ITM strategies is essential for sustainable tick management [16, 17].

## ACARICIDE ROTATION AND COMBINATION THERAPY

Acaricide rotation and combination therapy are two commonly employed strategies in ITM that aim to reduce resistance by minimizing selective pressure on tick populations. Acaricide rotation involves periodically changing the class of acaricide used to prevent ticks from developing resistance to any one specific chemical. This method is particularly effective for single-host ticks, such as *R. microplus*, which are exposed continuously to acaricides due to their close association with livestock throughout their lifecycle [6].

Combination therapy involves the simultaneous application of two or more acaricides with distinct modes of action to target different physiological pathways in ticks [25]. By exerting multiple selective pressures at the same time, this approach reduces the probability that individual ticks will survive treatment through single resistance mechanisms, thereby slowing the development and spread of acaricide resistance [22, 26]. Evidence indicates that combination therapy can be effective against both single-host and multi-host tick species when applied judiciously. For instance, the combined use of organophosphates and synthetic pyrethroids has demonstrated improved efficacy against *R. microplus*, limiting the rapid resistance development commonly observed with repeated use of a single acaricide class [6]. However, to maintain long term effectiveness, combination therapy should be implemented as part of an ITM framework, alongside strategic rotation, correct dosing, and non-chemical control measures [9].

## BIOLOGICAL CONTROL ALTERNATIVES

Biological control methods offer a promising non-chemical approach to tick management and are increasingly relevant in ITM strategies aimed at reducing acaricide reliance. Biological control agents, including entomopathogenic fungi, parasitoids, and predators, can naturally suppress tick populations without contributing to resistance. For instance, *Metarhizium anisopliae* and *Beauveria bassiana*, two fungi species pathogenic to ticks, have been studied for their ability to infect and kill multiple tick life stages, proving effective against both single-host and multi-host species [4, 11].

In East and Southern Africa, research on entomopathogenic fungi has shown that they can reduce tick populations in pastures where livestock graze. These fungi can be applied to areas frequented by ticks, such as cattle rest sites, where they persist in the soil and provide long term tick control. Unlike acaricides, fungi-based control is less likely to induce resistance because the fungi kill ticks through physical invasion rather than chemical pathways [24]. However, their effectiveness can vary based on environmental conditions, such as temperature and humidity, which affect fungal spore viability. Regions with high temperatures and low humidity may face challenges in maintaining fungal efficacy [13].

Biological control also includes introducing natural tick predators, such as chickens and certain bird species, which consume ticks from livestock pastures. On the other hand, these birds could further feed on cattle blood and wounds. While not a standalone solution, predators can significantly reduce tick populations and work synergistically with other ITM components. Moreover, some African pastoralists are reintroducing traditional practices, such as burning pastures to control tick populations, though this method has mixed ecological impacts and is not always suitable for modern grazing systems [15].

## INTEGRATED PASTURE AND HABITAT MANAGEMENT IN SEDENTARY AND PASTORAL SYSTEMS

Environmental control strategies, including pasture management and habitat modification, constitute an important pillar of ITM by reducing suitable tick habitats and interrupting key stages of the tick life cycle. Practices such as rotational grazing, pasture resting, and controlled stocking densities reduce host availability for ticks and can significantly lower infestation pressure. These approaches are particularly effective against multi-host tick species such as *A. variegatum*, which depend on repeated host contact and favorable microhabitats for survival and development across larval, nymphal, and adult stages [12].

In sedentary and semi-intensive livestock systems, pasture-based interventions can be systematically planned and implemented. Vegetation management around grazing areas, kraals, and watering points reduces humidity and shelter for off-host tick stages, thereby limiting tick survival. In some settings, carefully managed strategic pasture burning has been shown to reduce tick populations; however, this approach requires thorough ecological assessment to avoid soil degradation, biodiversity loss, and unintended disruption of ecosystem services [1].

Despite their demonstrated effectiveness, the applicability of pasture and habitat management strategies across much of SSA is limited by the predominance of extensive pastoral and transhumant production systems [27]. Communal land tenure arrangements, seasonal livestock mobility in response to rainfall and forage availability, and limited control over grazing intensity often make pasture burning, rotational grazing, and pasture resting impractical [11, 28]. In such systems, the inability to exclude livestock from specific grazing areas for extended periods reduces the effectiveness of habitat-based tick control measures [29].

Nevertheless, adapted, context-specific interventions remain feasible in pastoral settings. Localized habitat management around high-risk areas, such as communal kraals, watering points, and livestock routes, can reduce tick densities where animal aggregation is greatest [21]. Community-based coordination among pastoralists is particularly important, as isolated efforts by individual farmers are unlikely to yield sustained reductions in tick populations [20, 28]. When combined with strategic acaricide application, improved animal husbandry, and farmer education, these targeted pasture interventions can contribute meaningfully to overall ITM outcomes [17].

Evidence from humid, tick-prone regions of East Africa, including parts of Kenya and Uganda, indicates that integrating pasture management with judicious acaricide use can reduce tick burdens and slow the development of acaricide resistance [16]. However, successful implementation depends on tailoring interventions to local ecological conditions, production systems, and socio-economic realities. Consequently, pasture and habitat management should be viewed as complementary components of ITM rather than standalone solutions, particularly in pastoral landscapes where mobility and communal resource use constrain their full application [30, 31].

## EDUCATION AND FARMER PARTICIPATION

Education and farmer participation have a substantial effect on the adoption and implementation of new farming techniques. Oli *et al*. [32] reported that education improves the farming skills and productive ability of the farmer. Educating and training livestock farmers on the principles and benefits of ITM is essential for sustainable livestock farming. Farmers in SSA often lack access to training on acaricide application techniques, rotation schedules, and the biological mechanisms behind resistance [7]. Implementing ITM strategies effectively requires farmer understanding and participation, especially when it comes to adhering to protocols. Involvement of relevant stakeholders such as the veterinary pharmaceutical industry, government, and research institutions to enhance farmers’ knowledge on the importance of ITM through extension methods such as training and workshops could positively affect the farmers’ practices on tick control [31]. Understanding and active participation of farmers in trainings and workshops, particularly in adhering to rotation protocols and accurately applying correct rates, could improve adoption of these techniques [11]. Educating farmers can also foster awareness of emerging acaricide resistance and encourage community-based approaches, where entire farming communities collaborate on ITM efforts to limit tick populations across a region [26].

## KNOWLEDGE GAPS AND FUTURE RESEARCH DIRECTIONS

The Rapid Tick Exposure Test (RaTexT®) is a new method for detecting acaricide resistance in cattle ticks. This test provides rapid results within 24 hours on the exposure of partially engorged adult ticks to a specially designed acaricide-impregnated matrix [8]. Testing and monitoring of various tick species across SSA is essential for sustainable farming, as this test has been conducted in three countries in Africa. Current knowledge gaps include insufficient studies on multi-host tick resistance, limited information on resistance among wildlife tick populations, and a need for regional monitoring programs that can track and address resistance as it emerges [12, 32].

Future research should focus on identifying alternative control strategies that can complement or replace chemical acaricides. These may include biological controls, such as entomopathogenic fungi, and acaricide-free integrated management approaches, such as habitat management and tick vaccines, which could offer more sustainable long term solutions [11]. Furthermore, monitoring and collaboration between researchers, stakeholders, industry, veterinarians, and local farmers will also be critical in advancing ITM and reducing acaricide resistance over the long term [13, 28].

## CONCLUSION

Acaricide resistance has emerged as a widespread and escalating challenge across SSA, affecting both single-host and multi-host tick species and undermining the effectiveness of conventional tick control strategies. The evidence synthesized in this review demonstrates that single-host ticks, particularly *R. microplus*, exhibit higher and more rapid resistance development due to continuous exposure to acaricides, while multi-host ticks such as *A. variegatum* and *R. appendiculatus* show emerging resistance patterns shaped by ecological adaptability and intermittent exposure. Resistance to major acaricide classes, including synthetic pyrethroids, organophosphates, and amidines, is now widely reported, driven by mechanisms such as target-site mutations and enhanced metabolic detoxification. Environmental factors, including temperature, humidity, vegetation, and climate change, further influence tick distribution and accelerate resistance dynamics, particularly in high-density livestock systems.

From a practical perspective, these findings highlight the urgent need to transition from sole reliance on chemical control toward ITM approaches. Strategies such as acaricide rotation, combination therapy, biological control using entomopathogenic fungi, pasture and habitat management, and farmer education can collectively reduce selection pressure and improve long term control outcomes. Strengthening farmer awareness and promoting correct application practices are especially critical in smallholder systems, where misuse of acaricides remains a key driver of resistance.

A major strength of this review lies in its integrated analysis of biological, environmental, and socio-management factors influencing acaricide resistance, providing a comprehensive understanding of resistance dynamics across diverse ecological settings. However, several limitations persist, including limited data on resistance patterns in multi-host ticks, insufficient information on the role of wildlife reservoirs, and a lack of standardized, region-wide surveillance systems.

Future research should prioritize the development of sustainable alternatives to chemical control, including vaccines, advanced biological agents, and precision-based management strategies. There is also a need for coordinated regional monitoring programs, improved diagnostic tools such as rapid resistance detection assays, and stronger collaboration among researchers, policymakers, and farming communities.

In conclusion, combating acaricide resistance in SSA requires a multidisciplinary and region-specific approach that integrates scientific innovation with practical farm-level interventions. The adoption of ITM, supported by continuous surveillance, education, and policy-driven support, is essential to ensure sustainable tick control, reduce the burden of TBDs, and safeguard livestock productivity in the face of evolving environmental and resistance challenges.

## AUTHORS’ CONTRIBUTIONS

MY, NN and LQ: Identified the research topic, developed methodology, and drafted the manuscript. ZM and ZTHM: Reviewed and edited the manuscript. All authors read and approved the final manuscript.
